# Lapatinib, Nilotinib and Lomitapide Inhibit Haemozoin Formation in Malaria Parasites

**DOI:** 10.3390/molecules25071571

**Published:** 2020-03-29

**Authors:** Ana Carolina C. de Sousa, Keletso Maepa, Jill M. Combrinck, Timothy J. Egan

**Affiliations:** 1 Department of Chemistry, Faculty of Science, University of Cape Town, Rondebosch 7701, South Africa; ana.desousa@uct.ac.za; 2 Department of Medicine, Division of Pharmacology, Faculty of Health Sciences, University of Cape Town, Observatory 7925, South Africa; MPXMAK002@myuct.ac.za (K.M.); jill.combrinck@uct.ac.za (J.M.C.); 3 Institute of Infectious Disease and Molecular Medicine, Wellcome Centre for Infectious Diseases Research in Africa, Faculty of Health Sciences, University of Cape Town, Observatory 7925, South Africa; 4 Institute of Infectious Disease and Molecular Medicine, University of Cape Town, Rondebosch 7701, South Africa

**Keywords:** β-haematin, haemozoin, virtual screening, docking, drug repurposing, *Plasmodium*, antimalarials, tyrosine kinase inhibitors

## Abstract

With the continued loss of antimalarials to resistance, drug repositioning may have a role in maximising efficiency and accelerating the discovery of new antimalarial drugs. Bayesian statistics was previously used as a tool to virtually screen USFDA approved drugs for predicted β-haematin (synthetic haemozoin) inhibition and in vitro antimalarial activity. Here, we report the experimental evaluation of nine of the highest ranked drugs, confirming the accuracy of the model by showing an overall 93% hit rate. Lapatinib, nilotinib, and lomitapide showed the best activity for inhibition of β-haematin formation and parasite growth and were found to inhibit haemozoin formation in the parasite, providing mechanistic insights into their mode of antimalarial action. We then screened the USFDA approved drugs for binding to the β-haematin crystal, applying a docking method in order to evaluate its performance. The docking method correctly identified imatinib, lapatinib, nilotinib, and lomitapide. Experimental evaluation of 22 of the highest ranked purchasable drugs showed a 24% hit rate. Lapatinib and nilotinib were chosen as templates for shape and electrostatic similarity screening for lead hopping using the in-stock ChemDiv compound catalogue. The actives were novel structures worthy of future investigation. This study presents a comparison of different in silico methods to identify new haemozoin-inhibiting chemotherapeutic alternatives for malaria that proved to be useful in different ways when taking into consideration their strengths and limitations.

## 1. Introduction

Malaria is an infectious tropical disease caused by five human-infecting species of *Plasmodium* parasites that remain a major public health problem with a severe socio-economic impact, despite recent successes in control and eradication. It is transmitted by female *Anopheles* mosquitoes that introduce parasites into the human bloodstream during a blood meal. In 2017, an estimated 219 million cases of malaria occurred worldwide. The continued development of parasite resistance to antimalarial medicines is one of the threats hampering progress in eradicating this disease [1].

The previously highly successful drug chloroquine (CQ), which is no longer recommended for the treatment of *P. falciparum* infections because of widespread resistance, has a mechanism of action based on inhibition of the parasite haem detoxification process. During its blood stage, when it resides within the red blood cell (RBC), the parasite digests host haemoglobin as a source of amino acids, in part for its own nourishment. As a result, it releases large quantities of the haemoglobin cofactor haem into its digestive vacuole (DV), which is detoxified by biocrystallisation as haemozoin or malaria pigment. This is a relatively inert and highly insoluble microcrystalline substance, prominent in the trophozoite and schizont forms of blood-stage malaria parasites. By inhibiting haemozoin formation, CQ, as well as some other quinoline drugs, increase the concentration of free haem, which likely kills the parasite by catalysing the formation of reactive oxygen species [2]. Theoretical models supported by experimental evidence obtained in abiotic media has led to a hypothesis that haemozoin inhibiting drugs act by direct binding to the haemozoin crystal [3,4,5].

Resistance to CQ is associated with a protein in the DV membrane, *P. falciparum* chloroquine resistance transporter (PfCRT), which promotes structure specific efflux and is not directly related to the therapeutic target, but rather lowers the concentration of CQ; and in some cases related quinolines in the DV [6]. Haemozoin inhibition thus remains a very attractive malaria drug target. Indeed, the haemoglobin digestion pathway was recently classified as having a high probability of delivering successful new drugs [7]. Finding new structurally diverse chemical entities that are haemozoin inhibitors is a strategy to overcome the resistance mechanism.

In the early part of the drug development pipeline, a technique that has gained popularity as a means to accelerate drug discovery is high throughput screening (HTS). This involves experimental screening of large libraries of compounds against either a whole organism or a specific biological target. While HTS has been successful in identifying many potential leads, the time and cost invested are high. As computational science has evolved, virtual screening (VS) has become progressively useful. Virtual screening offers a cheaper and faster way of narrowing down choices by using in silico methods to filter a given set of compounds to arrive at a subset with an improved probability of being biologically active [8,9,10].

Virtual screening methods are divided into two categories: structure-based methods and ligand-based methods. Structure-based virtual screening (SBVS) uses molecular docking to select compounds before testing them experimentally by prioritising those predicted to bind best to a given drug target [9]. Large virtual libraries of available, and purchasable compounds can be docked into a pharmacological receptor site with known structure. The compounds are scored based on the binding energy and predicted interactions with the target, and the top scored hits are then selected for further experimental assays. With docking studies, thousands of compounds can be evaluated for potential biological activity at lower cost and shorter time when compared to HTS [11,12]. Recently, we reported the first structure-based virtual screening using the β-haematin crystal as a target by applying a docking method. This made use of the ZINC15 database, a subset of which was searched for compounds with high binding affinity with the surface of the β-haematin crystal. A hit rate of 73% was found, showing a large enrichment over random screening. The benzoxazole moiety was found to be a promising scaffold for further derivatisation, showing intraparasitic haemozoin inhibition [13].

Ligand-based virtual screening (LBVS) is based on the concept that compounds similar to known active compounds have a higher probability of being active than unrelated compounds. Methods include pharmacophore match, quantitative structure-activity relationships (QSAR), and similarity search [14]. We have previously used Bayesian statistics as a tool to virtually screen compounds for predicted β-haematin inhibition and antimalarial activity. The model was trained with HTS data combined from several studies consisting of over 800 active and nearly 42,000 inactive compounds. The study demonstrated the ability of this machine learning approach to prioritise compounds for HTS. It was also used to rank known United States Food and Drug Administration (USFDA) approved drugs based on their probability of being β-haematin (synthetic haemozoin) inhibitors that are bioactive against *P. falciparum* [15].

In this study, we further investigated the USFDA approved drugs, starting with those that were highest ranked in the Bayesian model as potential β-haematin inhibitors bioactive against *P. falciparum* [15]. We now report their experimental β-haematin and parasite growth inhibition activities (IC50). We then evaluate the performance of the SBVS method that we recently reported [13], using the USFDA approved drugs. This compound set was screened for binding to the β-haematin crystal and the top ranked drugs tested in a β-haematin inhibition assay. In total, 21 drugs were tested for their ability to inhibit β-haematin formation.

Lapatinib, nilotinib, and lomitapide showed the best activity for inhibition of both β-haematin formation and parasite growth. As a consequence, they were chosen to conduct a cellular haem fractionation assay to measure free haem and haemozoin in cultured *P. falciparum* cells as a function of dose. This confirmed that all three drugs inhibit haemozoin formation in the parasite. Lapatinib and nilotinib were the most potent parasite growth inhibitors and were therefore chosen as starting points for a LBVS screen using the diverse in stock ChemDiv compound catalogue consisting of 1.6 million compounds. The screen was based on shape and electrostatic similarity and was set to search for chemically diverse compounds able to inhibit β-haematin formation, which could then be flagged for further investigation. Finally, 180 compounds were purchased and tested using a β-haematin inhibition assay. They showed an overall β-haematin inhibition hit rate of 6.9%, representing a significant enrichment over random HTS.

## 2. Results and Discussion

### 2.1. In Vitro Assays of the USFDA Approved Drugs

As noted above, in a previous study we used a virtual screening approach to discover *P. falciparum* growth inhibitors that also inhibit β-haematin formation. This was accomplished using Bayesian statistical models for both in vitro antimalarial and β-haematin inhibition activities. These models were used to screen 1510 USFDA approved drugs as a validation step. In the top ranked 2.1% of compounds were six known β-haematin inhibiting drugs that are clinical antimalarials as well as hydroxychloroquine and quinidine barbiturate [15]. In this previous study, we did not experimentally test these USFDA approved drugs. Within the top ranked 20 β-haematin inhibiting antimalarially active drugs were three clinical antimalarials with known β-haematin inhibiting activity. Of the remaining 17 drugs, nine were available for purchase and we have tested their ability to inhibit β-haematin formation in a detergent mediated assay (Table 1).

For this assay, the neutral detergent nonidet P-40 was selected as a catalyst since it has previously been used it in a HTS assay for β-haematin inhibition. It allows β-haematin to be synthesized in an extracellular environment from haematin under physiological conditions of pH and temperature [16,18,19]. The extent of inhibition is determined by colorimetrically measuring the unreacted haematin using pyridine, which forms a complex with haem but not with β-haematin [20]. All of the tested drugs showed β-haematin inhibitory activity, except for thiabendazole. This inactivity may relate to its small size, almost resembling a fragment. In a recent investigation of new benzimidazoles active against β-haematin formation and parasite growth, L’abbate et al. observed that fragments similar in size to thiabendazole were inactive when compared to their parent compounds that contained an extra aromatic ring system. A molecular docking study conducted to attempt to rationalise these findings indicated that the lack of activity of the smaller fragments was probably a result of fewer contacts with the crystal surface, even though the benzimidazole fragment was the key pharmacophore. The most active benzimidazoles had a higher number of π-stacking interactions with the β-haematin crystal [21].

Lapatinib, imatinib, nilotinib, gefitinib, and erlotinib exhibited notable β-haematin inhibition IC_50_ values, which fell below 100 μM (Table 1), in a range similar to that of clinical haemozoin inhibitors (52.0 μM for quinine and 22.0 μM for chloroquine) [22,23]. They have previously been reported to exhibit antimalarial activity as indicated in PubChem [15]. These drugs are used in cancer treatment and are known tyrosine kinase inhibitors. Protein kinases are enzymes involved in the cell life cycle and are upregulated, amplified, or mutated in cancer cells. They are also attractive drug targets for many infectious disease [24,25]. Given the success in developing drugs targeting human kinases, *Plasmodium* kinases are attractive targets and kinase inhibitors have been explored as a potential next generation of antimalarials [26]. Surprisingly, the tyrosine kinase family seems to be absent in the malaria parasite genome although a number of putative tyrosine kinase-like kinases (TKLs) have been found in *Plasmodium* species [24,27]. Imatinib for instance, identified before as an antimalarial, was found to prevent the phosphorylation of erythrocyte membrane band 3 by inhibiting an erythrocyte tyrosine kinase causing destabilization of the erythrocyte membrane required for parasite egress, leading to parasite entrapment and termination of the infection [28].

Lapatinib was first recognized as an antimalarial in an effort to find a faster way of developing new antimalarial agents, where a set of bio-active compounds were tested against *Plasmodium falciparum* blood stages. The set included drugs that have undergone clinical studies in other therapeutic areas, but not achieved approval, and a set of USFDA-approved drugs [29]. In previous studies, nilotinib and lapatinib had been found to be active against the erythrocytic asexual stage of the malaria parasite [30].

In our β-haematin inhibition assay, they were shown to exhibit the best IC_50_ values, superior to the well-known reference drug chloroquine (Table 1). This is a finding consistent with the observation of blood stage activity.

Within the bottom ranked compounds, we investigated four drugs to test the prediction of their inactivity against β-haematin formation (Table 1). Dantrolene, nitrendipine, nimodipine, and acenocumarol did not show any inhibitory activity, consistent with the fact that they had already been reported inactive against malaria parasites in PubChem [15].

Having conducted β-haematin inhibition assays, we went on to measure parasite growth inhibition activity against the chloroquine- and pyrimethamine-resistant *P. falciparum* K1 strain cultured in vitro, as well the CQ-sensitive NF54 strain, permitting calculation of the resistance index (RI; Table 1).

A highlight of the findings in Table 1 is that 6 out of 9 drugs brought about inhibition of parasite growth. Lapatinib, nilotinib, and lomitapide showed the best activity, with IC_50_ values falling in the nanomolar range. The other drugs showed weak parasite growth inhibition or no activity. The RI value for CQ was 18, whereas for the other drugs tested the RI was below 4, indicating minimal cross-resistance with chloroquine.

### 2.2. Structure-Based Virtual Screening Against the β-haematin Crystal

**Figure 1:**
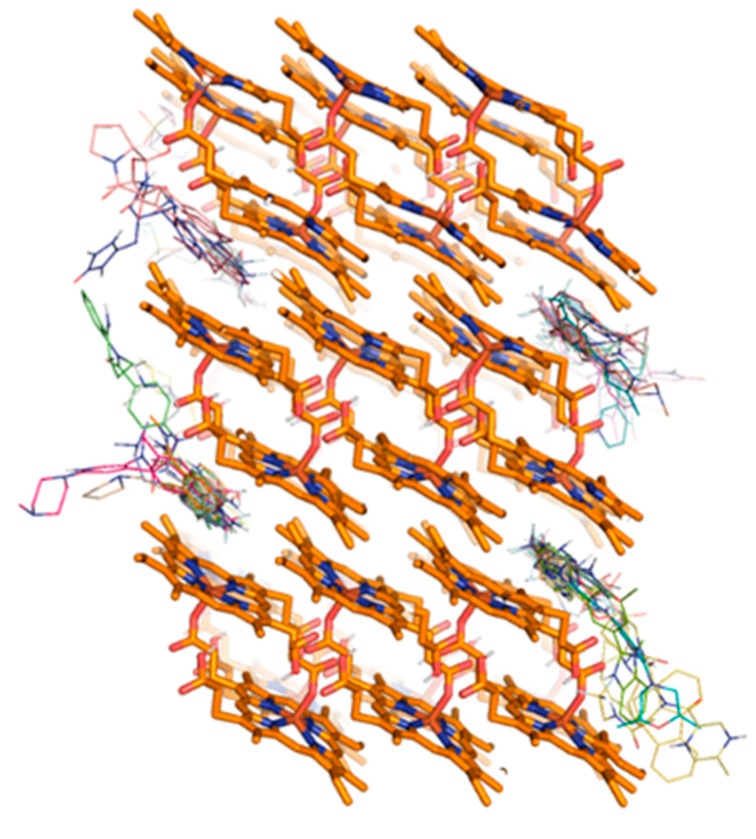
3D structure of the β-haematin crystal showing the top ranked drugs by Vina binding energy exhibiting a preference for the crevices within the [001] and [00$\bar{1}$] faces.

A serious disadvantage of the Bayesian classifier approach, as well as other machine learning methods, is the need for large numbers of inactives in the training set. These are seldom reported. This problem is in principle obviated in SBVS and given our recent success with this approach [13], we decided to investigate its success in screening the USFDA approved drugs. To find new β-haematin crystal growth inhibitors, we performed SBVS using the docking approach on the USFDA approved drug set available in the ZINC15 database containing 1607 drugs. ZINC15 is a public access database and tool set, initially developed to enable ready access to compounds for virtual screening, and ligand discovery as well as other cheminformatics applications [31]. Following energy minimization, the search for possible β-haematin crystal growth inhibitors by docking was performed with AutoDock Vina assembled in the PyRx Virtual Screening Tool [32]. The search space of the docking simulations covered all faces of the β-haematin crystal. This allowed the drugs to interact with any preferred surface. As previously observed [13], the top-ranked drugs showed a preference for the corrugations within the fastest-growing [001] face (Figure 1).

Once docking was completed, the drugs were selected from the top ranked compounds sorted by their Vina binding affinity (Supplementary Material Table S1) and visually inspected for favourable interactions such as π-π stacking, hydrogen-bonds, and electrostatic interactions with the crystal surface. Of these, 21 compounds were purchased for further investigation (Table 2).

Of the 21 drugs tested, five showed antimalarial and β-haematin inhibition activity. Imatinib, lapatinib, nilotinib, and lomitapide identified in the Bayesian model [15] were also found by the docking method; their IC_50_ values are repeated in Table 2. Telmisartan, on the other hand, was ranked solely by the SBVS method. Telmisartan has previously been reported to exhibit liver-stage activity of 25 nM [33]. Current findings indicated blood stage activity via haemozoin inhibition as suggested by inhibition of β-haematin crystallisation as well as trophozoite growth inhibition (Table 2).

While the anticancer agents lapatinib and nilotinib have been reported to have antimalarial activity, no similar activity has previously been reported for lomitapide to our knowledge. Lomitapide is clinically used to treat homozygous familial hypercholesterolaemia through the inhibition of the microsomal triglyceride transfer protein and may be especially interesting for drug repositioning [34].

Drug repositioning or drug repurposing is a strategy for identifying new applications of approved drugs. It may have a role maximising efficiency and reducing costs in the discovery of new antimalarial drugs. This strategy has a lower risk of failure, since the drug has already been proved safe in preclinical models and in humans [35,36]. Our study has also provided mechanistic insights into the antimalarial action of lapatinib, nilotinib, and lomitapide, demonstrating that they are haemozoin inhibitors. With the continued loss of antimalarials to resistance, new chemotherapeutic alternatives will be needed in the coming years [37].

It should be noted that panobinostat, rolapitant, netupitant, sonidegib, and carbozantinib were not available for purchase. Panobinostat has been reported in PubChem to be strongly active against malaria parasites with an IC_50_ 4 nM and 8–45 fold selectivity for the parasite over human neonatal foreskin fibroblast or human embryonic kidney cells [38]. Rolapitant and netupitant are antiemetic drugs and have not yet been reported to exhibit antimalarial activity.

The rate of false positives obtained in this study using SBVS against the β-haematin crystal target was considerably higher than that which we recently reported using a portion of the ZINC virtual compound library [13]. A possible explanation may be found in the fact that most drugs contain aromatic rings that can π-stack with the flat haem rings in haemozoin. It should also be noted that while the strongest interaction is predicted to be with the [001] face, this does not necessarily imply that no interaction occurs with other faces. In a recent investigation of benzimidazole haemozoin inhibitors, we found that not only docking to the [001] face of β-haematin but also to the second fastest growing [011] face had to be considered to explain the observed activity [21]. The absence of these additional binding modes may play a role in reducing the accuracy of the model, as may the absence of solvents and lack of steps and kinks at the surface of the crystal, which are known to represent sites of drug binding [4]. Nonetheless, despite this false positive rate, the model still had a 24% hit rate, far higher than that found in HTS using the β-haematin inhibition assay [16,19,39,40,41,42]. Furthermore, the SBVS method correctly identified the three most important β-haematin inhibiting drugs identified in the USFDA set using the Bayesian model, namely lapatinib, nilotinib, and lomitapide. On the other hand, it has to be admitted that the Bayesian model [15] for the USFDA approved drug set performed better than the SBVS model applied in this investigation.

### 2.3. Haem Fractionation Assay

To establish whether the most active β-haematin inhibiting parasite active drugs actually inhibit haemozoin formation in the parasite, lapatinib, nilotinib and lomitapide were chosen for investigation using a cellular haem fractionation assay [43]. This experiment allows the measurement of free haem and haemozoin fractions in cultured *P. falciparum* cells.

**Figure 2:**
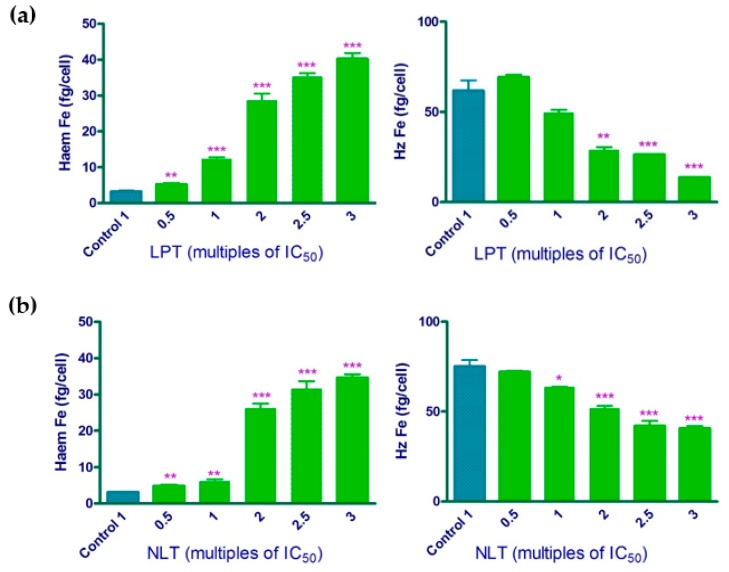

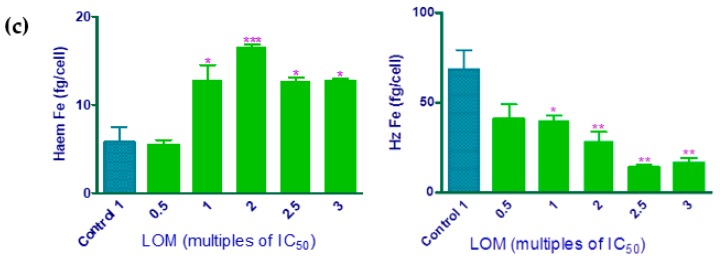
Freely exchangeable haem (left panels) and haemozoin (Hz, right panels) expressed as fg haem iron per trophozoite in isolated *P. falciparum* trophozoites following 32 h incubation with (**a**) lapatinib (LPT), (**b**) nilotinib (NLT), and (**c**) lomitapide (LOM) at multiples of their IC_50_. Control: untreated cells. * *p* < 0.05; ** *p* < 0.01; *** *p* < 0.001.

Lapatinib, nilotinib, and lomitapide were all found to increase the freely exchangeable haem, and decrease haemozoin in a dose dependent manner confirmed by an unpaired two tailed t-test relative to control, therefore confirming that these drugs inhibit cellular haemozoin formation (Figure 2). The increase in free haem is believed to be responsible for parasite killing, even though the detailed mechanism of how free haem kills the parasite is still a subject of investigation [2].

### 2.4. Ligand-Based Virtual Screening Using Lapatinib and Nilotinib as Templates for Shape and Eletrostatic Similarity

The large number of commercial screening libraries now available enables a focus on exploration of structural diversity. This is particularly relevant in antimalarial drug discovery considering the prevalence of resistance. A convenient option is to select a diversity-oriented library, such as one of those currently offered by various vendors. To identify new β-haematin formation inhibitors, we chose the ChemDiv in-stock diverse collection database containing 1,535,478 compounds (downloaded in March 2019).

The compounds were imported into DataWarrior [44] and subjected to toxicity and drug-likeness predictions in order to filter out non drug-like and potentially toxic compounds. Toxicity was predicted by calculating the risk of a compound having mutagenic, tumorigenic, reproductive, or irritant effects. This reduced the size of the library to 715,239 compounds. This was followed by conformer generation in DataWarrior [44]. DataWarrior is an open-source tool that offers, among its various applications, the prediction of physicochemical properties, data preparation, and analysis with interactive and dynamic visualization for cheminformatics calculations.

Rapid Overlay of Chemical Structures (ROCS) [45] was chosen to first look for compounds with shape and chemical similarities to lapatinib and nilotinib. The shape essentially consists of the volume and conformation of the template molecule which is used for alignment, while the chemical similarity considers the position of hydrogen-bond donors, hydrogen-bond acceptors, hydrophobes, negative charges, positive charges, and rings [45,46,47].

The compounds in the library were separately screened in ROCS using lapatinib and nilotinib as templates, respectively. Thereafter, they were ranked by Tanimoto Combo that uses shape alignment (shape Tanimoto score criterion) and the color Tanimoto score (color alignment). The 7200 (approximately 1%) best ranked compounds as indicated by ROCS were then used as input for analysis using the EON (Electrostatic Similarity for Lead-Hopping) software package [9]. ROCS aligns the molecules to provide a better input to the EON program. EON can be used to screen chemical libraries for electrostatic similarity to a lead compound permitting lead-hopping. The input molecules need to already be aligned for this; thus, EON uses output structures from programs such as ROCS, leading to compounds with similar shape and electrostatics [9].

The 80 best-ranked molecules obtained from each of the lapatinib and nilotinib (Supplementary Material Table S2) similarity searches were selected and purchased for experimental testing. The compounds were tested in the nonidet P-40 mediated assay for β-haematin inhibition [16,18,20] at a fixed concentration of 150 μM. For both the lapatinib and nilotinib series, 6 hits each were found showing β-haematin activity (their IC_50_s are reported in Table 3), giving a hit rate of 7.5% in both cases.

When tested in the NF54 strain of P. falciparum (Table 3), the lapatinib series led to only one hit with weak activity (IC_50_ 4.91 ± 0.44 μM), while the nilotinib series gave five hits (best IC_50_ 3.84 ± 0.33 μM). Thus, it is clear that SBVS using the β-haematin crystal gives better hit rates than the LBVS method reported here. Nonetheless, the method has the strategic advantage of hopping from one drug-like molecular scaffold to another, allowing rapid discovery of new chemotypes and thus suggesting useful new hits for further exploration in drug discovery [46,47,48]. Indeed, the structures identified in this study with β-haematin inhibition and/or parasite killing activity might be of interest for future investigations.

## 3. Materials and Methods

### 3.1. Compounds

All the tested drugs were purchased from Merck Chemicals (Germiston, South Africa). Inhibitors of β-haematin formation predicted by LBVS were purchased from ChemDiv (San Diego, CA, USA).

### 3.2. Detergent Mediated Assay for β-haematin Inhibition

The β-haematin inhibition assay was performed based on the method described by Carter et al. and Sandlin et al. [16,18] in 96-well plates. The pyridine-ferrochrome method developed by Ncokazi and Egan was used to measure unreacted haematin [20]. The UV-vis absorbances on the plate was read at 405 nm on a Thermo Scientific Multiskan GO plate reader (Thermo Scientific, Waltham, MA, USA). The sigmoidal dose-response curves were plotted using GraphPad Prism version 6 (GraphPad Software, Inc., La Jolla, CA, USA) to calculate the IC_50_ of each compound.

### 3.3. Plasmodium Lactate Dehydrogenase (pLDH) Assay

Culturing of parasites followed the earlier method by Trager and Jensen [49] and the pLDH assay was based on that of Makler et al. [50] The K1 strain of P. falciparum (chloroquine and pyrimethamine resistant) and the CQ-sensitive PfNF54 strain were used to test in vitro antimalarial activity. The assay is based on detecting the presence of P. falciparum lactate dehydrogenase activity in trophozoites after 48 h incubation in a 96-well plate. The IC_50_ values were obtained using non-linear dose-response curve fitting analysis via GraphPad Prism v 5.0.0 software (GraphPad Software Inc., La Jolla, CA, USA).

### 3.4. Structure-based Virtual Screening against the β-haematin Crystal

To identify new β-haematin formation inhibitors, the ready-to-dock 3D structures of the USFDA approved drugs were obtained from the ZINC15 database (fetched March 2018).

The compounds were imported into OpenBabel within the Python Prescription Virtual Screening Tool (PyRx) [32] and subjected to energy minimisation. This was performed in multiple rounds with the Universal Force Field (UFF) using the conjugate gradient algorithm. The total number of steps was set to 2000 and number of steps for update set to 1. In addition, the minimisation was set to stop at an energy difference of less than 0.01 kcal/mol.

Structure- based virtual screening applying docking simulations was performed using the AutoDock Vina tool compiled in PyRx (Scripps Research Institute, San Diego, CA, USA) [32]. Here, the structure of the β-haematin crystal previously published was used as the macromolecule (receptor) [51]. The search space encompassed the whole of the modelled crystal (made up of 27 unit cells) with the following size in Å: centre (x, y, z) = (18, 20, −14), dimensions (x, y, z) = (45, 51, 42). The docking simulation was then run at an exhaustiveness of 8 and set to only output the lowest energy pose. The docked poses with β-haematin were imported into PyMOL Molecular Graphics System to be visually inspected [52].

### 3.5. Haem Fractionation Assay

Target validation for lapatinib, nilotinib, and lomitapide was carried out via a cell fractionation assay, optimized to a multi-well colorimetric assay for determining haem species in *P. falciparum* as described by Combrinck et al. [43]. The cellular fractionation allows for direct quantification of the three major haem species in isolated trophozoites namely; haemoglobin, freely exchangeable haem, and haemozoin. Target validation is evaluated by measuring the increase in freely exchangeable haem and the decrease in haemozoin formation.

### 3.6. Statistical Analysis

A two-tailed t-test (95% CI) was used for determination of statistical significance of differences in measurements relative to controls and is displayed using asterisks on graphs (* *p* < 0.05; ** *p* < 0.01; *** *p* < 0.001). The data represent a minimum of three repeats with standard deviations calculated for each of the average results. All the analysis was done using GraphPad Prism version 5.0.0 software.

### 3.7. Ligand-based Virtual Screening

The Chemdiv in-stock diverse collection database containing 1,535,478 compounds (downloaded March 2019) was imported in DataWarrior [44] software for in silico toxicity and drug-likeness filtering. Still in DataWarrior (openmolecules.org, Allschwil, Switzerland), conformers for each one of the compounds were then generated using the random, low energy bias algorithm, and the MMFF94s force field. The structures of nilotinib and lapatinib selected as query molecules were built using ChemDraw 3D Ultra (15.1.0.144, CambridgeSoft) to draw their three-dimensional structure. Then they were also submitted to the same conformer generation method and their lowest energy conformation was chosen for comparison with each of the molecules in the prepared database.

ROCS (version 3.3.2.2, OpenEye Scientific Software, free for noncommercial use) used lapatinib and nilotinib as templates to virtually screen the given set of compounds according to structural features and molecular volumes of query compounds, observing the maximum overlap with respect to the shape which is approximated by atom-centered overlapping Gaussians and used to calculate the maximal intersection of the volume of two molecules. In addition to the three-dimensional shape superposition, ROCS can consider chemistry alignment, known as ‘color’ used in a chemical force field (color force field- CFF) to measure chemical complementarity, and to refine shape comparison based on chemical similarity. It is composed of SMARTS that define chemical centers, and rules to determine how such centers interact. The ImplicitMillsDean CFF selected for this study define six similar TYPE color force-fields. The types are hydrogen-bond donors, hydrogen-bond acceptors, hydrophobes, anions, cations, and rings [45,46,47]. Ranked by Tanimoto Combo (shape Tanimoto score criterion plus color Tanimoto score), 7200 hit compounds were then used as input for EON (version 2.3.2.2, OpenEye Scientific Software) [9]. EON was used to compare the electrostatic potential maps using Tanimoto scores to the pre-aligned lapatininb and nilotinib molecules by ROCS, re-ranking the hit compounds by matching their eletrostatic maps with the template molecule.

## 4. Conclusions

In this work, we experimentally confirmed the accuracy of our previously reported Bayesian model of β-haematin inhibiting anti-*Plasmodium* active drugs from the USFDA approved list by showing that 89% of the purchased compounds (8/9) from the available top 20 compounds that excluded the quinolines were active in inhibiting β-haematin formation. This would rise to 93% (13/14) if the quinolines on this list were included. The hit rate for activity against the parasite was almost as impressive, with 67% (6/9) showing some activity and three having nanomolar activity.

Finally, this study has permitted a direct comparison between different in silico methods for identifying new haemozoin inhibiting compounds active against *P. falciparum*. The machine learning Bayesian classification approach proved to be the most successful. The hit rate for SBVS was much lower at 24% (5/21) for both β-haematin and parasite growth inhibition, although still far above that for random screening. Notably, it still succeeded in identifying the three most active compounds and has the clear advantage of not requiring large quantities of prior data. LBVS, proved to be the least successful, with a hit rate of 7.5% for β-haematin inhibition, but only 3.1% for parasite inhibition. Nonetheless, the actives do have novel structures worthy of further investigation. Thus, although there is a clear hierarchy of efficiency, all three methods are useful in different ways, provided their limitations are taken into consideration.

## Figures and Tables

**Table 1:** Inhibition of β-haematin formation and parasite activity in CQ-sensitive (*Pf*NF54) and multidrug-resistant (*Pf*K1) strains of *P. falciparum* of top-ranked USFDA approved drugs selected on the basis of their ranking in a previously published Bayesian model [15].

Bayesian Rank	Drug	β-haematin IC_50_ (μM)	*Pf*NF54 IC_50_ (µM)	*Pf*K1 IC_50_ (µM)	RI ^2^
1	Lapatinib	5.43 ± 0.03	0.26 ± 0.07	0.85 ± 0.02	3.3
2	Amodiaquine	21 ^4^	NT ^3^	0.0086 ^5^	-
3	Imatinib	99 ± 5	3.9 ± 0.1	NT ^3^	-
4	Nafarelin	48 ± 5	NA ^1^	NT ^3^	-
5	Nilotinib	7 ± 1	0.28 ± 0.02	0.38 ± 0.06	1.4
6	Antrafenine	NT ^3^	NT ^3^	NT ^3^	-
7	Vapreotide	48 ± 7	NA ^1^	NT ^3^	-
8	Gefitinib	20.4 ± 0.8	11.5 ± 0.4	NT ^3^	-
13	Thiabendazole	> 500	NA ^1^	NT ^3^	-
14	Erlotinib	17.6 ± 0.2	13.3 ± 0.4	NT ^3^	-
19	Lomitapide	68.2 ± 0.9	0.56 ± 0.10	0.69 ± 0.14	1.2
21	Chloroquine	26 ± 0.3	0.015 ± 0.001	0.272 ± 0.005	18
1505	Dantrolene	>500	NT ^3^	NT ^3^	-
1506	Nitrendipine	>500	NT ^3^	NT ^3^	-
1508	Nimodipine	>500	NT ^3^	NT ^3^	-
1510	Acenocumarol	>500	NT ^3^	NT ^3^	-

^1^ not active at tested concentration of 10 µg/mL; ^2^ Resistance Index (RI) = *Pf*K1-IC_50_/*Pf* NF54-IC_50_. ^3^ Not tested. ^4^ Data from Sandlin et al. [16]. ^5^ Data from Guglielmo et al. [17].

**Table 2:** The SBVS top-ranked FDA approved drugs and their inhibition of β-haematin formation and parasite activity in the CQ-sensitive (*Pf*NF54) strain of *P. falciparum*.

Zinc Code	Binding Affinity	Drug	β-haematin IC_50_ (μM)	*Pf*NF54 IC_50_ (µM)
zinc000001550499	−13.4	Cinacalcet	NA ^1^	-
zinc000052716421	−12.9	Flibanserin	NA ^1^	-
zinc000000601229	−12.3	Azelastine	NA ^1^	-
zinc000012503187	−12.3	Conivaptan	NA ^1^	-
zinc000022010649	−12.2	Panobinostat	- ^2^	-
zinc000003816514	−12	Rolapitant	- ^2^	-
zinc000003784182	−12	Adapalene	NA ^1^	-
zinc000003978005	−12	Dihydroergotamine	NA ^1^	-
zinc000019632618	−12	Imatinib	99.0 ± 4.76	3.9 ± 0.14
zinc000000968263	−11.9	Cyclobenzaprine	NA ^1^	-
zinc000003816287	−11.9	Axitinib	NA ^1^	-
zinc000027990463	−11.8	Lomitapide	68.2 ± 0.85	0.56 ± 0.10
zinc000006716957	−11.8	Nilotinib	7.3 ± 1.2	0.28 ± 0.02
zinc000001530977	−11.8	Naftifine	NA ^1^	-
zinc000011681563	−11.8	Netupitant	- ^2^	-
zinc000000025958	−11.7	Rucaparib	NA ^1^	-
zinc000000538312	−11.6	Risperidone	NA ^1^	-
zinc000068202099	−11.6	Sonidegib	NA ^1^	-
zinc000003776633	−11.5	Acrivastine	NA ^1^	-
zinc000003798734	−11.5	Acitretin	NA ^1^	-
zinc000000968264	−11.5	Cyproheptadine	NA ^1^	-
zinc000000001261	−11.4	Desloratadine	NA ^1^	-
zinc000001550477	−11.4	Lapatinib	5.43 ± 0.03	0.26 ± 0.07
zinc000003939013	−11.3	Fosaprepitant	NA ^1^	-
zinc000070466416	−11.2	Cabozantinib	- ^2^	-
zinc000001530886	−11.2	Telmisartan	51.07 ± 0.67	4.99 ± 1.7
zinc000019144231	−7.4	(S)-Chloroquine ^3^	- ^2^	-
zinc000019144226	−8	(R)-Chloroquine ^3^	- ^2^	-
-	-	(±)-Chloroquine ^3^	26 ± 0.26	0.015 ± 0.001

^1^ not active at tested concentration of 150 μM. ^2^ Not in stock at the time of the study. ^3^ CQ is available and tested as a racemate, but for docking, a specific enantiomer must be constructed. We therefore considered both enantiomers, which were docked separately.

**Table 3:** The LBVS hits and their structures followed by experimental β-haematin formation inhibition and parasite growth inhibition IC_50_ data in the CQ-sensitive (*Pf*NF54) strain of *P. falciparum*.

Rank EON	Chemdiv Code	Structure	β-haematin IC_50_ (μM)	*Pf*NF54 IC_50_ (µM)
Query molecule	Lapatinib	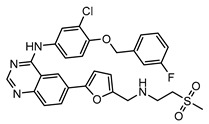	5.43 ± 0.03	0.26 ± 0.07
17	F424-1065	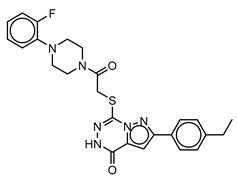	353.9 ± 59.96	4.91 ± 0.44
24	C514-0466	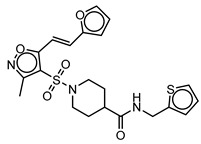	143.55 ± 32.88	NA ^1^
27	C514-0233	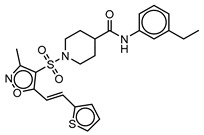	254.8 ± 59.26	NA ^1^
48	G637-3522	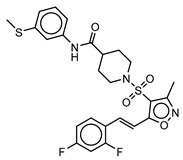	290.05 ± 42.07	NA ^1^
54	F731-0128	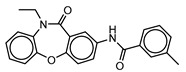	207.15 ± 19.02	NA ^1^
62	C514-0129	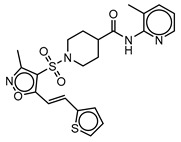	183.5 ± 4.67	NA ^1^
Query molecule	Nilotinib	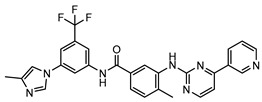	7.3 ± 1.2	0.28 ± 0.02
2	F871-0652	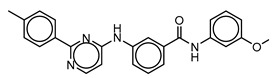	156.15 ± 1.63	6.42 ± 0.57
16	F871-0649	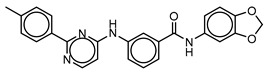	373.4 ± 98.0	8.09 ± 0.12
42	F725-0689	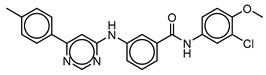	85.73 ± 40.41	3.9 ± 0.14
79	G416-5459	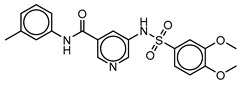	100.95 ± 22.85	NA ^1^
80	D444-0908	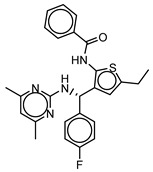	68.25 ± 6.41	3.84 ± 0.33
113	F545-1038	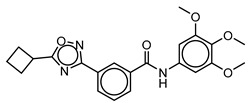	243.9 ± 11.46	NA ^1^

^1^ not active at tested concentration of 10 µg/mL.

## Supplementary Materials

The following are available online, Table S1: The top 4% FDA-approved drugs classified by the SBVS and their binding affinity with the surface of β-haematin crystal, Table S2: The ChemDiv purchased top ranked compounds classified by EON rank and their smiles codes.
