# Supplementary material for: Lapatinib, Nilotinib and Lomitapide Inhibit Haemozoin Formation in Malaria Parasites
Source: Molecules. 2020 Mar 29;25(7):1571. doi: 10.3390/molecules25071571 (PMC7180468; doi:10.3390/molecules25071571)
Supplement: Supplementary file 1 [file molecules-25-01571-s001.pdf]

molecules

Supplementary Material

Lapatinib, nilotinib and lomitapide inhibit haemozoin formation in malaria parasites

Ana Carolina C. de Sousa [1], Keletso Maepa [2], Jill M. Combrinck [2,4] and Timothy J. Egan [1,3,*]

1 University of Cape Town, Faculty of Science, Department of Chemistry, Rondebosch 7701, South Africa; ana.desousa@uct.ac.za
2 University of Cape Town, Department of Medicine, Division of Pharmacology, Faculty of Health Sciences, Observatory 7925, South Africa;
3 University of Cape Town, Institute of Infectious Disease and Molecular Medicine, Rondebosch 7701, South Africa; timothy.egan@uct.ac.za
4 Wellcome Centre for Infectious Diseases Research in Africa, Institute of Infectious Disease and Molecular Medicine; jill.combrinck@uct.ac.za
* Correspondence: timothy.egan@uct.ac.za

Table S1. The top 4% FDA approved drugs classified by the SBVS and their binding affinity with the surface of β-haematin crystal.

Zinc code	Binding affinity	Drug	Zinc code	Binding affinity	Drug
zinc000001550499	-13.4	Cinacalcet	zinc000011679756	-11	Eltrombopag
zinc000052716421	-12.9	Flibanserin	zinc000000608382	-11	Flavoxate
zinc000000601229	-12.3	Azelastine	zinc000000006157	-10.9	Epinastine
zinc000012503187	-12.3	Conivaptan	zinc000001895505	-10.9	Bazedoxifene
zinc000022010649	-12.2	Panobinostat	zinc000100070954	-10.9	Glimepiride
zinc000003816514	-12	Rolapitant	zinc000004175630	-10.8	Orap
zinc000003784182	-12	Adapalene	zinc000003823475	-10.8	Eletriptan
zinc000003978005	-12	Dihydroergotamine	zinc000052955754	-10.8	Ergotamine
zinc000019632618	-12	Imatinib	zinc000002570895	-10.8	Celebrex
zinc000000968263	-11.9	Cyclobenzaprine	zinc000003874185	-10.7	Mefloquine
zinc000003816287	-11.9	Axitinib	zinc000000004351	-10.7	Alaway
zinc000027990463	-11.8	Lomitapide	zinc000000001148	-10.7	Doxepin
zinc000006716957	-11.8	Nilotinib	zinc000000075126	-10.7	Zofran
zinc000001530977	-11.8	Naftifine	zinc000007997897	-10.7	Naftin
zinc000011681563	-11.8	Netupitant	zinc000000057522	-10.6	Ting
zinc000000025958	-11.7	Rucaparib	zinc000000001370	-10.6	Estazolam
zinc000000538312	-11.6	Risperidone	zinc000030691797	-10.6	Perampanel
zinc000068202099	-11.6	Sonidegib	zinc000029416466	-10.6	Sqv
zinc000003776633	-11.5	Acrivastine	zinc000000002272	-10.6	Ketoprofen
zinc000003798734	-11.5	Acitretin	zinc000000004893	-10.5	Asenapine
zinc000000968264	-11.5	Cyproheptadine	zinc000001530625	-10.5	Dronabinol
zinc000000001261	-11.4	Desloratadine	zinc000000968257	-10.5	Amitriptyline
zinc000001550477	-11.4	Lapatinib	zinc000003786192	-10.5	Sulindac
zinc000003939013	-11.3	Fosaprepitant	zinc000001539579	-10.5	Targretin

zinc000070466416	-11.2	Cabozantinib	zinc000000000740	-10.5	Restoril
zinc000001530886	-11.2	Telmisartan	zinc000000006427	-10.5	Diazepam
zinc000051951647	-11	Panobinostat	zinc000000002299	-10.5	Asenapine
zinc000003831151	-11	Montelukast	zinc000006745272	-10.5	Stivarga
zinc000027428713	-11	Aprepitant	zinc000000537931	-10.4	Loratadine
zinc000003831332	-11	Phylloquinone	zinc000095616937	-10.4	Dasabuvir
zinc000000004785	-11	Carbamazepine	zinc000043207238	-10.4	Canagliflozin
zinc000043206370	-11	Niraparib	zinc000000000323	-10.4	Flurbiprofen

Table S2. The Chemdiv purchased top ranked compounds classified by the EON rank and their smiles code

EON rank	Molecule Name	Smiles
Query molecule	Lapatinib	O=S(CCNCC1=CC=C(C2=CC=C3N=CN=C(NC4=CC=C(OCC5=CC=CC(F)=C5)C(Cl)=C4)C3=C2)O1)(C)=O
1	V018-2342	Cc(cc1)ccc1NC(c1csc(-c(cccc2)c2OCc(cc2)ccc2F)n1)=O
2	2028-0102	CCOC(C([C@@H](c1ccccc1)N1C2=O)=C(C)N=C1S/C2=C\c(cccc1)c1OCc(cccc1)c1Cl)=O
3	G637-0552	CC(c1cccc(NC(C(CC2)CCN2S(c2c(/C=C/c3ccc(C)cc3)onc2C)(=O)=O)=O)c1)=O
4	S558-0318	O=C(Nc1cccc(F)c1)N(CC1)C[C@@H]1Oc(cccc1)c1-c1nc(-c2nccnc2)no1
5	G637-0483	Cc1noc(/C=C/c2ccc(C)cc2)c1S(N(CC1)CCC1C(Nc(cccc1)c1F)=O)(=O)=O
6	Z250-1328	O=C([C@H](CCC1)CN1C(NC1=O)=Nc2c1scc2-c1ccccc1)NCc1cnccc1
7	2029-0102	CCOC(C([C@@H](c(cc1)ccc1OC)N1C2=O)=C(C)N=C1S/C2=C\c(cccc1)c1OCc(cccc1)c1Cl)=O
8	L227-0004	Cc(cc1)ccc1-c1nn2c(N(CC3)CCC3C(Nc3cccc(C(F)(F)F)c3)=O)cc(C)nc2c1
9	5648-2232	O=C(CN(C(/C(/S1)=C/c(cccc2)c2OCc2cc(F)ccc2)=O)C1=O)N1CCOCC1
10	Z250-1285	O=C([C@H](CCC1)CN1C(NC1=O)=Nc2c1scc2-c1ccccc1)NCCC1=CCCCC1
11	M347-0486	Cc1csc(-c2cc(S(Nc(cccc3)c3F)(=O)=O)cn2CC(N2CCOCC2)=O)n1
12	Z250-1934	O=C([C@H](CCC1)CN1C(NC1=O)=Nc2c1scc2-c1ccccc1)N1CCCC1
13	C514-0440	Cc1noc(/C=C/c2ccco2)c1S(N(CC1)CCC1C(Nc1cc(SC)ccc1)=O)(=O)=O
14	L520-0507	Cc1cc(C(N(CSC2)[C@@H]2C(NCc2csc(-c3cccs3)n2)=O)=O)cc(C)c1
15	M347-0487	Cc1csc(-c2cc(S(Nc3cc(F)ccc3)(=O)=O)cn2CC(N2CCOCC2)=O)n1
16	Z250-2563	Cc1cccc(-c2csc(C(N3)=O)c2N=C3N(CC2)CCC2C(NCC=C)=O)c1
17	F424-1065	CCc(cc1)ccc1-c(cc12)nn1C(SCC(N(CC1)CCN1c(cccc1)c1F)=O)=NNC2=O
18	D356-2238	Cc1cccc(CSCCNC(C(CC2)CCN2S(Cc(c(F)ccc2)c2Cl)(=O)=O)=O)c1
19	C928-0456	COc(ccc(-c1nc(CCNS(c2cc(CCCC3)c3cc2)(=O)=O)cs1)c1)c1OC
20	Z250-1085	CC(C)NC(C(CC1)CCN1C(NC1=O)=Nc2c1scc2-c(cccc1)c1F)=O
21	Z250-2571	Cc1cccc(-c2csc(C(N3)=O)c2N=C3N(CC2)CCC2C(NCCC2=CCCCC2)=O)c1
22	Z250-1343	O=C([C@H](CCC1)CN1C(NC1=O)=Nc2c1scc2-c1ccccc1)NC[C@@H]1OCCC1
23	F061-1291	Cc1noc(/C=C/c2c(C)ccc(C)c2)c1S(N(CCC1)C[C@H]1C(NCc1ncccc1)=O)(=O)=O
24	C514-0466	Cc1noc(/C=C/c2ccco2)c1S(N(CC1)CCC1C(NCc1cccs1)=O)(=O)=O
25	5762-1653	O=C(/C(/N1)=C\c2cn(Cc3ccccc3)c3c2cccc3)N(Cc(cccc2)c2F)C1=O

26	SA04-0326	Cc1ncc(COCC2CC2)c([C@H](CCC2)CN2C(Cc2cc(OC)ccc2)=O)n1
27	C514-0233	CCc1cccc(NC(C(CC2)CCN2S(c2c(/C=C/c3cccs3)onc2C)(=O)=O)=O)c1
28	2028-0099	CCOC(C([C@H](c1ccccc1)N1C2=O)=C(C)N=C1S/C2=C\c(cccc1)c1OCc(cc1)ccc1Cl)=O
29	V003-3993	COc(cc1)cc([C@H](CN(C2)C(c3cccc(Cl)c3)=O)[C@H]2C(NCc2ncccc2)=O)c1OC
30	S581-0950	COc(cccc1)c1N(CC1)CCN1C(C[C@@H](CCCCN1Cc2ccccc2)C1=O)=O
31	V015-2163	CCC(CC)C(Nc(cc1)ccc1-c1nc(-c(cc2)ccc2OC)ccc1)=O
32	S544-0192	Cc1cc(C)nn1Cc1ccc(C(N2C[C@H](CCOc(cccc3)c3F)CC2)=O)o1
33	K906-3779	Cc1noc(/C=C/c2cccs2)c1S(N(CC1)CCC1C(N1CCCCCC1)=O)(=O)=O
34	C776-4109	Cc1c(Cn2c(N(CC3)CCC3C(Nc3cc(OC)cc(OC)c3)=O)nc3c2cccc3)cccc1
35	S606-1398	O=C(C(C=C1)=NNC1=O)N(CC1)C[C@H]1N(c1ncccc1N1CC2CCCCC2)C1=O
36	L227-0081	CC(C)c(cc1)ccc1NC(C(CC1)CCN1c1cc(C)nc2cc(-c3ccc(C)cc3)nn12)=O
37	Z250-1127	NC(CNC(C(CC1)CCN1C(NC1=O)=Nc2c1scc2-c(cccc1)c1F)=O)=O
38	G637-0592	Cc1noc(/C=C/c2ccc(C)cc2)c1S(N(CC1)CCC1C(Nc(cc1)cc(Cl)c1F)=O)(=O)=O
39	G237-0141	Cc1nc(ccc(S(N(CC(Nc(cc2)cc3c2OCO3)=O)Cc2ccccc2)(=O)=O)c2)c2s1
40	C514-0464	C[C@H](CCC1)[C@H](C)[C@@H]1NC(C(CC1)CCN1S(c1c(/C=C/c2ccco2)onc1C)(=O)=O)=O
41	M136-0585	Cc(cc1)ccc1-c1noc(-c2cccnc2N(CC2)CCC2C(NCCCOC)=O)n1
42	Z250-1650	CC[C@H](C)NC([C@H](CCC1)CN1C(NC1=O)=Nc2c1scc2-c(cc1)cc(OC)c1OC)=O
43	L520-0422	O=C(COc1ccccc1)N(CSC1)[C@@H]1C(NCc1csc(-c2ccccc2)n1)=O
44	V022-7995	O=C(c1csc(-c2cccc(OCc(cc3)cc(Cl)c3Cl)c2)n1)Nc1ccccc1
45	E222-0034	COC(c1ccc(-c2ccco2)n1CC(N1CCC(Cc2ccccc2)CC1)=O)=O
46	1123-0326	O=C(CSc1nnnn1-c1ccccc1)N/N=C/c(cc1)ccc1Br
47	C890-0334	C[C@@H](C(NCc1ncccc1)=O)n(c(C)c1C=NN2c3ccccc3)c(C)c1C2=O
48	G637-3522	Cc1noc(/C=C/c(ccc(F)c2)c2F)c1S(N(CC1)CCC1C(Nc1cc(SC)ccc1)=O)(=O)=O
49	V020-5238	COc(cccc1)c1-c(cc1)cc2c1oc([C@H](CCC1)N1C(C1CCCC1)=O)n2
50	8013-5505	Cc1c(-c2c[nH]c3c2cccc3)nc(-c(cc2)cc(OC)c2OC)s1
51	SC13-0036	O=C(COc(cc1)ccc1F)N(CCC1)C[C@@H]1N(Cc1cnccc1)C(C1CCC1)=O
52	G682-0058	Cc(cc1C)cc(C)c1NC(CN(N=C(c1ccccc1)c1c2cccc1)S2(=O)=O)=O
53	Z250-1086	CC[C@H](C)NC(C(CC1)CCN1C(NC1=O)=Nc2c1scc2-c(cccc1)c1F)=O
54	F731-0128	CCN(c(cccc1)c1Oc(cc1)c2cc1NC(c1cc(C)ccc1)=O)C2=O
55	L227-1125	Cc1cc(-c2nn3c(N(CC4)CCC4C(Nc(cc4)ccc4F)=O)cc(C)nc3c2)cc(C)c1
56	D400-0082	COc1c(CNC(COc2cc(-c3nc(-c4ccccc4)no3)ccc2)=O)cccc1
57	G883-0692	Cc1cc(C)nn1Cc1ccc(C(NCc(cc2)cc(CC3)c2N3C(c2ccccc2)=O)=O)o1
58	G420-0186	Cc(cc1)ccc1-c1nn(ccnc2SCC(N(CC3)CCN3c(cc3)ccc3F)=O)c2c1
59	F758-0175	CCOc1c(CNC([C@@H](CCC2)CN2c2nc3ncccc3n3c2ccc3)=O)cccc1
60	S558-0161	O=C(Nc(cccc1)c1F)N(CC1)C[C@H]1Oc(cccc1)c1-c1nc(-c2ccccc2)no1
61	L437-0064	O=C(NCCc1ccccc1)Nc(cccc1)c1-c1cc(cccc2)c2[nH]1
62	C514-0129	Cc1noc(/C=C/c2cccs2)c1S(N(CC1)CCC1C(Nc1ncccc1C)=O)(=O)=O
63	L227-0209	Cc1cccc(CNC(C(CC2)CCN2c2cc(C)nc3cc(-c(cc4)ccc4Cl)nn23)=O)c1
64	V001-1009	O=C(Nc1ccccc1)N(CCC1)[C@H]1c1nc(cc(cc2)-c(cccc3)c3F)c2o1

65	L227-0264	Cc1nc2cc(-c(cc3)ccc3Cl)nn2c(N(CC2)CCC2C(NCCc2ccco2)=O)c1
66	Z250-0045	CCOC([C@H](CCC1)CN1C(NC1=O)=Nc2c1scc2-c1ccc(C)cc1)=O
67	L227-1012	Cc1nc2cc(-c3ccccc3)nn2c(N(CC2)CCC2C(N[C@H](CCC2)c3c2cccc3)=O)c1
68	SA57-0395	Cc1c(Cn2nccc2)c(C(N(CC2)CCC2(C2)OCC[C@@H]2Oc2nccnc2)=O)no1
69	L227-0187	Cc1nc2cc(-c(cc3)ccc3Cl)nn2c(N(CC2)CCC2C(NCc2ccco2)=O)c1
70	F113-0158	Cc(cc1)ccc1-c1nc2cccnc2n1CCNC(Nc1cccc(SC)c1)=O
71	Z250-1271	CC[C@@H](C)NC([C@@H](CCC1)CN1C(NC1=O)=Nc2c1scc2-c1ccccc1)=O
72	Z250-0354	O=C1NC(N(CC2)CCN2c(cccc2)c2F)=Nc2c1scc2-c1cc(F)ccc1
73	E756-0628	Cn(c(cc1)c2cc1OC)c(C(NCCc(cccc1)c1OC)=O)c2N(CCC1)C1=O
74	G637-0718	CCCN(C(C(CC1)CCN1S(c1c(/C=C/c2ccc(C)cc2)onc1C)(=O)=O)=O)c(cccc1)c1F
75	Z250-1136	O=C(C(CC1)CCN1C(NC1=O)=Nc2c1scc2-c(cccc1)c1F)N[C@H](CC1)c2c1cccc2
76	Y050-0064	CN(CC(Nc(cc1)cc2c1OCO2)=O)S(c(cc(cc1)OC)c1OC)(=O)=O
77	Z250-1648	CCCNC([C@H](CCC1)CN1C(NC1=O)=Nc2c1scc2-c(cc1)cc(OC)c1OC)=O
78	1185-0223	Cc1c(-c(cc2)ccc2OC)nc(Nc2ccc(C)cc2)s1
80	G664-0290	O=C(c(sc1ncccc11)c1-n1cccc1)NCCc1ccco1
Query molecule	Nilotinib	CC1=CN(C2=CC(NC(C3=CC(NC4=NC=CC(C5=CC=CN=C5)=N4)=C(C=C3)C)=O)=CC(C(F)(F)F)=C2)C=N1
1	L775-0627	Cn1nc(COCc2cc(F)ccc2)c(C2)c1CCN2C(Nc1cccc(SC)c1)=O
2	F871-0652	Cc(cc1)ccc1-c1nccc(Nc2cccc(C(Nc3cc(OC)ccc3)=O)c2)n1
3	2688-0031	O=C(c1cccc(Nc2nc(-c3ccccc3)c(cc(cc3)Cl)c3n2)c1)Nc(cc1)ccc1Cl
4	Y041-7161	O=C(c1cc(Nc2ncccn2)ccc1)NCCc1ccco1
5	L775-0534	Cc1ccc(COCc2nn(C)c(CC3)c2CN3C(Nc2cc(C)ccc2)=O)cc1
6	S926-7975	O=C(CCn1nccc1)N(CCC1)C[C@H]1OCc1noc(-c2cnccc2)n1
7	L775-0642	Cn1nc(COCc2cccc(F)c2)c(C2)c1CCN2C(Nc(cc1)ccc1F)=O
8	F871-0684	Cc(cc1)ccc1-c1nccc(Nc2cccc(C(Nc(cc3)ccc3Cl)=O)c2)n1
9	F517-0033	COc1cccc(C(Nc2cc(-n3nnnc3)ccc2)=O)c1
10	8020-1623	O=C(c1cnccc1)Nc(cccc1)c1-c1nc(-c2cnccc2)no1
11	G404-2528	CCn1cnc(S(N(CCC2)C[C@@H]2C(Nc(cc(cc2)OCC)c2OCC)=O)(=O)=O)c1
12	F545-1102	Cc(ccc(NC(c1cc(-c2noc(C3CCC3)n2)ccc1)=O)c1)c1F
13	E715-0019	COc(ccc(Cl)c1)c1NC([C@H](CCC1)CN1c1ncnc2c1nc1n2CCCCC1)=O
14	C875-0226	Cc1ccc(CSc2nc(ccnc3)c3n2CC(Nc2cc(SC)ccc2)=O)cc1
15	SB20-0299	O=C(c1cn(C[C@@H](CCC2)N2C(c2ncccn2)=O)nn1)NCc1ccccc1
16	F871-0649	Cc(cc1)ccc1-c1nccc(Nc2cccc(C(Nc(cc3)cc4c3OCO4)=O)c2)n1
17	G756-0949	CC(C=CN12)=CC2=NC(COc(cccc2)c2NC(Nc(cc2)cc(Cl)c2F)=O)=CC1=O
18	G756-0096	COc1cccc(NC(Nc(cccc2)c2OCC(N=C2N3C=CC=C2)=CC3=O)=O)c1
19	G404-0917	CCc1cccc(NC([C@H](CCC2)CN2S(c2cn(C)cn2)(=O)=O)=O)c1
20	D400-2258	Cc1noc(-c2cccc(NC(c3cc(OC)cc(OC)c3)=O)c2)n1
21	F517-0037	O=C(c1cccc(Cl)c1)Nc1cc(-n2nnnc2)ccc1
22	C875-0228	CCN(C(Cn1c(SCc2ccc(C)cc2)nc2c1cncc2)=O)c1cc(C)ccc1
23	G266-0449	CCN(c(cc(c(N1CCCCC1)c1)NC(Nc(cccc2)c2OCC)=O)c1N1CC)C1=O
24	Y031-2577	CCC(Nc1cccc(C(Nc2cc(C)cc(C)c2)=O)c1)=O

25	D223-0599	CCOC(c1c(NC(C(C(C=C2)=O)=NN2c2cc(C)ccc2)=O)sc(C(C)=O)c1C)=O
26	F305-0378	Cc(cc1)ccc1-c(cc1)nnc1N(CCC1)C[C@H]1C(NCCc(cc1)ccc1Cl)=O
27	L150-0763	CCc(cc1)ccc1C(Nc1cc(-n2nnnc2)ccc1)=O
28	C301-7073	CN(c(cc(c(N1CCCC1)c1)NC(Nc(cccc2)c2OC)=O)c1N1C)C1=O
29	F321-0593	Cc(ccc(C(Nc1cc(-c2noc(C3CCCC3)n2)ccc1)=O)c1)c1NC(c1ccco1)=O
30	F517-0024	COc1cc(C(Nc2cc(-n3nnnc3)ccc2)=O)cc(OC)c1
31	V019-7709	Cc(cc1)cc(NC(c2ccccc2)=O)c1N(CCCN1Cc(cc2)ccc2Cl)C1=O
32	6228-2472	Cc1cc(NC(c2ccc(COc(cccc3)c3Br)o2)=O)cc(C)c1
33	G266-0623	Cc(cc1)cc(NC(Nc(c(N2CCCC2)c2)cc(N3C)c2N(C)C3=O)=O)c1OC
34	S926-1888	COCc1nc(CO[C@H](CCC2)CN2C(Cc2ccccc2)=O)no1
35	G609-0057	Cc1cc(NC(c2cc3c(-c4ccccc4)onc3cc2)=O)cc(C)c1
36	M735-1560	Cc1noc(C)c1CC(N1C[C@@H](COc(cccc2)c2C(N2CCCC2)=O)CCC1)=O
37	3346-1206	COc1cc(C(Nc(cccc2)c2-c2nc(cccc3)c3s2)=O)cc(OC)c1
38	F758-0012	O=C([C@@H](CCC1)CN1c1nc2ncccc2n2c1ccc2)NCc1ccccc1
39	F517-0306	Cc(cccc1)c1C(Nc(cc(cc1)-n2nnnc2)c1N1CCCCC1)=O
40	V028-8720	Cc(cc1)cc(NC(Nc2ccccc2)=O)c1N(CCCN1Cc(cccc2)c2F)C1=O
41	F545-0213	Cc1nc(-c2cccc(C(Nc3cc(C)ccc3)=O)c2)no1
42	F725-0689	Cc(cc1)ccc1-c1cc(Nc2cccc(C(Nc(cc3)cc(Cl)c3OC)=O)c2)ncn1
43	M373-1454	O=C(Cc1cc(F)ccc1)N(CCC1)C[C@H]1c1ccnc(Nc(cccc2)c2F)n1
45	P634-0305	CN(CCc1ccncc1)C(CC[C@H](CCC1)CN1c1ncnc(NC2CC2)c1)=O
46	C301-4436	O=C(c1cc(Nc2nc(cccc3)c3n3c2nnc3)ccc1)NCCc1ccccc1
47	J077-1053	C[C@@H](c1cccc(NC(c2cccc(N(CCCN3Cc(cccc4)c4F)C3=O)c2)=O)c1)O
48	F545-1038	COc1cc(NC(c2cc(-c3noc(C4CCC4)n3)ccc2)=O)cc(OC)c1OC
49	D699-0125	CCOC([C@@H](CCC1)CN1C(Cn1c2nc(-n3nc(C)cc3C)nnc2c2c1cccc2)=O)=O
50	F720-1196	CCOC(c1cccc(NC([C@@H](CCC2)CN2C(C=C2)=NN(c3cccc(Cl)c3)C2=O)=O)c1)=O
51	V022-7328	Cc(cc1)cc(NC(c(cc2)ccc2OC)=O)c1N(CCCN1Cc2c(C)cccc2)C1=O
52	D444-0755	Cc1cc([C@H](c(cc2)ccc2Cl)Nc2nc(C)cc(C)n2)c(NC(c2ccccc2)=O)s1
53	G609-0354	Cc(cc1)ccc1-c1c(cc(cc2)C(Nc3cc(C)cc(C)c3)=O)c2no1
54	C155-0612	C[C@H](CC1)CC[C@@H]1C(N[C@H](Cc1ccccc1)C(NCc1ncccc1)=O)=O
55	P181-1188	Cc1cc(C(Nc2c(-c3nc(-c4cc(F)ccc4)no3)scc2)=O)cc(C)c1
56	L829-0029	CC(C)[C@H](c1nc(-c2cccc(-c3csc(C)n3)c2)no1)NC(c1ccco1)=O
57	F545-0059	Cc1nc(-c2cccc(C(NCc3ccco3)=O)c2)no1
58	L361-0965	Cc1cc(NC(Nc2c(CCc3nc(cccc4)c4n3C)cccc2)=O)cc(C)c1
59	6466-1310	CCOc(cccc1)c1NC(c(cnn1c(C(F)F)c2)c1nc2-c1ccccc1)=O
60	Y042-7013	CSCC[C@@H](c1nc(cccc2)c2[nH]1)NC(c1cc(Nc2ncccn2)ccc1)=O
61	F431-0588	CN(c(c(N1C)c2)cc(NC(c(cc3)cc(OC)c3OC)=O)c2Sc(cccc2)c2Cl)C1=O
62	G609-0017	COc1cc(NC(c2cc3c(-c4ccccc4)onc3cc2)=O)cc(OC)c1OC
63	Y200-4626F	Cc1cc(C(Nc2cccc(C(NCc3ccccc3)=O)c2)=O)no1
64	G609-0485	CCc1cccc(NC(c2cc3c(-c4ccc(C)cc4)onc3cc2)=O)c1
65	P188-1446	COc1cc(NC(CN(C=Cc2c3n(Cc4cccc(F)c4)cc2)C3=O)=O)cc(OC)c1
66	G856-0108	O=C(c1cc(F)ccc1)Nc(cccc1)c1-c1nc(cccc2)c2s1

67	M337-0336	CCc1nnc(-c2cc(n(CC(NCc3ncccc3)=O)cc3)c3cc2)o1
68	J077-0953	Cc1cccc(CN(CCCN2c3cc(C(NCc4ncccc4)=O)ccc3)C2=O)c1
69	C326-0168	C[C@@](C[C@H]1N2)(N(c3cc(C(Nc(cc4)ccc4OC)=O)ccc3)C2=O)Oc2c1cccc2OC
70	P806-0813	O=C(c1cocc1)N[C@@H](CCC1)CN1c1cc(-c(cc2)ccc2F)n[nH]1
71	F177-0089	COc1cc(NC(c(cccc2)c2-c2nc(-c3ccccc3)no2)=O)cc(OC)c1OC
72	G786-1401	CSc(cc1)ccc1C(Nc1nc(-c2ccncc2)cs1)=O
73	F779-1163	Cn1c(cccc2)c2nc1CCc1cccc(NC(c2ccccc2)=O)c1
74	D103-1046	Cc(cc1)cc2c1OC(C(Nc1c(C(c3ccccc3)=O)oc3c1cccc3)=O)=CC2=O
75	Y031-2590	CCC(Nc1cccc(C(Nc2cc(OC)ccc2)=O)c1)=O
76	D223-0622	Cc1cccc(N(C=C2)N=C(C(Nc3c(C(NC[C@@H]4OCCC4)=O)c(CCC4)c4s3)=O)C2=O)c<br>1
77	C201-0252	C[C@@](C[C@H]1N2)(N(c3cc(C(Nc(cc4OC)cc(OC)c4OC)=O)ccc3)C2=O)Oc2c1cccc2
78	L539-0085	COc(cc1)ccc1Oc1ccnc(-c2cccc(C(NCc(cc3)ccc3Cl)=O)c2)n1
79	G546-0546	COc1cc(NC(c2cc(-c3ncccc3)nc3ccccc23)=O)cc(OC)c1OC
80	G416-5459	Cc1cccc(NC(c2cc(NS(c(cc3)cc(OC)c3OC)(=O)=O)cnc2)=O)c1
